# Improving Adherence to High-Dose Antipsychotic Therapy Guidelines in a Community Mental Health Team: A Closed-Loop Audit

**DOI:** 10.1192/bjo.2026.11826

**Published:** 2026-06-30

**Authors:** Samrat Prasai, Bianca Olayefelix, Shafaq Sajid, Peter Kelsall

**Affiliations:** Pennine Care Foundation Trust, Manchester, United Kingdom

## Abstract

**Aims:**

Patients requiring off-label treatment with high-dose antipsychotic therapy (HDAT) are at greater risk of metabolic, cardiovascular and extrapyramidal side effects. Close monitoring is essential to ensure safety. Pennine Care Foundation Trust’s (PCFT) guidelines for the initiation and monitoring of HDAT are divided into 8 domains: (1) consultant initiation, (2) patient consent procedures, (3) blood tests, (4) ECG monitoring, (5) annual clinic review, (6) identification of reported side effects, (7) GP notification and (8) HDAT form completion. 100% compliance is required in all domains. This audit evaluates whether a HDAT register improves adherence to initiation and monitoring requirements.

**Methods:**

A closed-loop two-cycle audit of patients at a PCFT general adult community mental health team service was conducted by reviewing medical records to identify HDAT patients using an online medication regimen analysis tool. A retrospective baseline audit was undertaken in December 2024 assessing adherence to local PCFT guidelines on HDAT initiation and monitoring. A HDAT register was created locally based on these findings requiring biannual review by resident doctors. A re-audit with identical methodology was conducted in January 2026.

**Results:**

2.13% and 2.06% of patients received HDAT in the baseline audit and re-audit respectively. Baseline audit confirmed 60.6% compliance with local guidelines on average across all domains. Following implementation of a HDAT register and biannual review, compliance increased to an average of 77%. Compliance rose in 5 domains with 100% compliance in domains 2, 5, 6 and 7. Compliance increased from 0% to 67% in domain 8. Compliance remained at 83% in domain 1 across both audits. There was a 25.4% and 75.1% relative reduction in compliance with blood tests (domain 3) and ECG monitoring (domain 4) respectively due to inconsistencies in patient engagement and in requesting physical health tests on initiation of HDAT. Notably, HDAT was discontinued in 50% of patients identified in the baseline audit following review of the HDAT register.

**Conclusion:**

The HDAT register improved compliance with guidelines in 5 out of 8 domains. Registration enabled review of indication for continued therapy leading to treatment cessation in 50% of the initial cohort. The register improved record keeping with HDAT form completion rising to 67%. Biannual review of the register highlighted the need for further improvements in completing blood tests and ECGs. Solutions involve issuing all required tests as patient held request forms in standardised bundles prior to initiation, in addition to increasing review of the register to quarterly and improving coordination with primary care.

